# Prevalence of mental disorders in refugees and asylum seekers: a systematic review and meta-analysis

**DOI:** 10.1017/gmh.2022.29

**Published:** 2022-06-14

**Authors:** Martina Patanè, Samrad Ghane, Eirini Karyotaki, Pim Cuijpers, Linda Schoonmade, Lorenzo Tarsitani, Marit Sijbrandij

**Affiliations:** 1 Department of Clinical, Neuro-, and Developmental Psychology and WHO Collaborating Center for Research and Dissemination of Psychological Interventions, Vrije Universiteit, Amsterdam, The Netherlands; 2 Parnassia Psychiatric Institute, The Hague, The Netherlands; 3 Medical Library, Vrije Universiteit, Amsterdam, The Netherlands; 4 Department of Human Neurosciences, Sapienza University of Rome, Rome, Italy

**Keywords:** Mental disorders, meta-analysis, prevalence, refugees

## Abstract

**Background:**

Studies have identified high rates of mental disorders in refugees, but most used self-report measures of psychiatric symptoms. In this study, we examined the percentages of adult refugees and asylum seekers meeting diagnostic criteria for major depressive disorder (MDD), post-traumatic stress disorder, bipolar disorder (BPD), and psychosis.

**Methods:**

A systematic literature search in three databases was conducted. We included studies examining the prevalence of MDD, post-traumatic stress disorder, BPD, and psychosis in adult refugees according to a clinical diagnosis. To estimate the pooled prevalence rates, we performed a meta-analysis using the Meta-prop package in Stata (PROSPERO: CRD42018111778).

**Results:**

We identified 7048 records and 40 studies (11 053 participants) were included. The estimated pooled prevalence rates were 32% (95% CI 26–39%; *I*^2^ *=* 99%) for MDD, 31% (95% CI 25–38%; *I*^2^ *=* 99.5%) for post-traumatic stress disorder, 5% (95% CI 2–9%; *I*^2^ = 97.7%) for BPD, and 1% (95% CI 1–2%; *I*^2^ *=* 0.00%) for psychosis. Subgroup analyses showed significantly higher prevalence rates of MDD in studies conducted in low-middle income countries (47%; 95% CI 38–57%, *p =* 0.001) than high-income countries studies (28%; 95% CI 22–33%), and in studies which used the Mini-International Neuropsychiatric Interview (37%; 95% CI 28–46% *p =* 0.05) compared to other diagnostic interviews (26%; 95% CI 20–33%). Studies among convenience samples reported significant (*p* = 0.001) higher prevalence rates of MDD (35%; 95% CI 23–46%) and PTSD (34%; 95% CI 22–47%) than studies among probability-based samples (MDD: 30%; 95% CI 21–39%; PTSD: 28%; 95% 19–37%).

**Conclusions:**

This meta-analysis has shown a markedly high prevalence of mental disorders among refugees. Our results underline the devastating effects of war and violence, and the necessity to provide mental health intervention to address mental disorders among refugees. The results should be cautiously interpreted due to the high heterogeneity.

## Introduction

During the past decade, the trend of global displacement has been growing (UNHCR, 2020). In 2020, the United Nations High Commissioner for Refugees (UNCHR) estimated that more than 11 million people have been displaced throughout the year, and the proportion of this population has been continued to rise, despite the COVID-19 pandemic and closure of borders (UNHCR, 2020). Although refugees (people who, owing to a well-founded fear of persecution, are forced to escape from his or her country) and asylum seekers (people seeking protection from persecution or serious harm in a country other than their own) are defined in different ways (UNHCR, 2020), both groups may have been forced to face various stressors, such as persecution, violence, torture, detention, and the loss of homes and livelihoods. Such traumatic events may result in persistent mental health problems and overall decreased functioning (Steel *et al*., 2009; Marquez, 2016).

During the last years, several studies have examined the prevalence of mental disorders in refugees and asylum seekers. However, the resulting prevalence rates in this population present great variability (Fazel *et al*., 2005; Porter and Haslam, 2005; Bogic *et al*., 2015; Foo *et al*., 2018; Morina *et al*., 2018; Charlson *et al*., 2019; Blackmore *et al*., 2020), ranging from 5% (Fazel *et al*., 2005) to 80% (Bogic *et al*., 2015) for depression, from 4% (Charlson *et al*., 2019) to 88% (Morina *et al*., 2018) for PTSD, and from 1.5% (Blackmore *et al*., 2020) to 2% (Fazel *et al*., 2005) for psychosis. A systematic review on the prevalence of serious mental disorders in refugees resettled in high-income western countries reported weighted average rates of 5% for major depressive disorder (MDD), 9% for posttraumatic stress disorder (PTSD) and 2% for psychotic disorders (Fazel *et al*., 2005). A meta-analysis among refugees and asylum seekers residing outside their country of origin reported a prevalence of 31.5% for PTSD, of 31.5% for depression, and 1.5% for psychosis (Blackmore *et al*., 2020). Another meta-analysis that examined the prevalence of mental disorders in conflict settings showed estimated rates of 22.1% for any mental disorder (13% for depression and 4% for PTSD) (Charlson *et al*., 2019). The variability across studies could be explained by differences in the origins and background of the analysed populations, the sample size, the sampling methods, and the diagnostic tools used (e.g. self-report, semi-structured or structured clinical interview) (Westermeyer and Janca, 1997; de Jong *et al*., 2003; Fazel *et al*., 2005; Bogic *et al*., 2015; Giacco *et al*., 2018; Giacco and Priebe, 2018; Charlson *et al*., 2019). Furthermore, the nature of the displacement, the context of the emergency, as well as the aspects of the host country/environment are additional aspects to consider in the variability of these studies (Giacco and Priebe, 2018).

Many epidemiological studies use self-report instruments to estimate the prevalence of mental disorders in refugees because they are easily administered and cost effective. However, such instruments overestimate prevalence rates considerably (Domken *et al*., 1994; Fazel *et al*., 2005; Steel *et al*., 2009). In fact, self-report instruments have been shown to overestimate true PTSD-rates by a factor of about 3.5 (Engelhard *et al*., 2007). Similar results have been also shown in depression studies (Domken *et al*., 1994; Steel *et al*., 2009; Krebber *et al*., 2014). One of the reasons is that many self-report instruments are designed for quickly picking-up mental disorders while minimising false negatives, therefore cut-offs are set with high sensitivity. Therefore, they identify more patients who may have a mental disorder than those diagnosed with clinician-administered interviews (Thombs *et al*., 2018). Another reason could be that self-report tools usually do not take functional impairment due to symptoms into account (McKnight and Kashdan, 2009). Besides, translations of existing self-report instruments may not adequately measure psychiatric symptoms across cultures (Hunt and Bhopal, 2004). Finally, overestimation may occur as a consequence of the so-called *over-endorsement bias,* the tendency of respondents to generously claim different types of symptoms on a checklist (Kroenke, 2001). Thus, more rigorous instruments, such as clinical diagnostic interviews, are more appropriate to estimate the prevalence of mental health disorders in refugees and asylum seekers (Fazel *et al*., 2005; Steel *et al*., 2009). In addition, clinicians' experience in using structured interviews can increase the reliability of symptoms measurement and psychiatric diagnoses even among samples with different cultural backgrounds (Alarcon *et al*., 1999; Aboraya *et al*., 2006). Nevertheless, among the structured and semi-structured interviews there may also be some differences in accuracy. For example, studies have shown that the Mini-International Neuropsychiatric Interview – MINI, which has been designed as a briefer and quicker diagnostic screen than other interviews (Sheehan *et al*., 1997) identified more people as depressed than the Composite International Diagnostic Interview – CIDI, and the Structured Clinical Interview – SCID (Levis *et al*., 2018; Levis *et al*., 2019; Wu *et al*., 2021).

In the present study, we aimed to estimate the prevalence of diagnoses of serious mental disorders [MDD, PTSD, bipolar disorder (BPD), and psychosis] in refugees and asylum seekers from conflict-affected areas. Serious mental disorders are conditions resulting in serious functional impairment, which interferes with major life activities (NIH, 2019). Other definitions (Slade *et al*., 1997) take into account the duration and the disability they produce, for example in terms of the disability they are associated with. Within the Global Burden of Disease (GBD) study, the top-three mental disorders associated with the highest level of disability are MDD, BPD and psychosis (Kronenberg *et al*., 2017; James *et al*., 2018). Although not included in the GBD, PTSD is particularly relevant for refugees and asylum seekers since refugees may be exposed to multiple traumatic events, including interpersonal violence that may cause severe disability (Palic *et al*., 2016).

Similar to previous studies (Fazel *et al*., 2005; Blackmore *et al*., 2020), we included only studies that employed diagnostic interviews. Further, our study differs from previous studies in that we were able to include a larger number of studies than before since the number of studies increased during the past years. In addition, we were able to examine the prevalence of less common disorders such as BPD. Finally, due to the increased number of included studies, we could perform additional subgroup analyses, such as comparing the prevalence of serious mental disorders between refugees resettled in low-middle-income *v.* high-income countries, and among different kind of population sample (convenience samples *v.* probability-based samples).

## Method

### Search strategy and selection criteria

We report our meta-analysis according to Preferred Reporting Items for Systematic Reviews and Meta-Analysis (PRISMA) statement (www.prisma-statement.org) (Moher *et al*., 2009) (see online Supplementary materials).

The present study was registered in PROSPERO on 5^th^ December 2018 under the number: CRD42018111778.

A comprehensive search was performed in the bibliographic databases PubMed, Embase.com and APA PsycInfo (via Ebsco) from inception to June 4, 2020, by a medical librarian. Search terms included controlled terms (MeSH in PubMed, Emtree in Embase and PsycINFO thesaurus terms) as well as free text terms. The following terms were used (including synonyms and closely related words) as index terms or free-text words: ‘refugees’ and ‘mental illnesses’. A search filter was used to limit the results to adults. The search was performed without date or language restrictions. Duplicate articles were excluded. The full search strategies for all databases can be found in the online Supplementary materials.

Studies were considered eligible for this meta-analysis if they examined (a) the prevalence of serious mental disorders (MDD, PTSD, BPD, and psychosis), (b) in an adult (⩾18 years) population of refugees and asylum seekers who had to cross their country borders, (c) according to the criteria of Diagnostic and Statistical Manual of Mental Disorders (DSM III, IV, or 5) or International Classification of Diseases (ICD 9 or10) (d) assessed with a structured or semi-structured clinical interview, such as the Structured Clinical Interview – SCID, Mini-International Neuropsychiatric Interview – MINI, or Composite International Diagnostic Interview – CIDI. No cut-off has been considered to evaluate the diagnosis' severity. Disorders have been evaluated serious according with the National Institute of Mental Health (NIH) and GBD considerations (Kronenberg *et al*., 2017; James *et al*., 2018; NIH, 2019).

The definitions of refugees (a person who, owing to a well-founded fear of persecution, is forced to escape from his or her country) and asylum seekers (a person who seeks protection from persecution or serious harm in a country other than their own) were in agreement with the UNHCR Master Glossary of Terms (UNHCR, 2006) and Asylum and Migration Glossary 6.0 (Network, 2018). No restrictions were applied to the origin of the studies. Cross-sectional studies, follow-up studies, cohort data studies and register-based studies were eligible. To reduce possible selection bias, we excluded studies conducted on samples selected in psychiatric services and concerning internally displaced people (IDPs) (UNHCR, 2006).

All title and abstracts were screened by two researchers independently (MP/MS or SG). We retrieved the full texts of all abstracts that seemed eligible for inclusion. Subsequently, we performed a full-text selection according to our pre-specified eligibility criteria. Disagreements between the reviewers were resolved by discussion.

### Data extraction

For every eligible study, two reviewers independently (MP/MS or SG) extracted data related to the year of publication, the country of data collection, the country of the refugees' origins, the sample size of the population, the age, the gender, the time elapsed as refugees or asylum seekers, the diagnostic tool used. Finally, we extracted the prevalence rates of MDD (current episode and recurrent), PTSD, BPD and psychotic disorder. In the absence of absolute numbers and if allowed by the data reported, the percentages of prevalence rates were converted into numbers. The time since displacement was reported in three different categories: (1) people who have spent more than five years as refugees or asylum seekers (⩾5), (2) people who have spent between 5 years and 1 year as refugees or asylum seekers (>1 < 5) and (3) people who have spent less then 1 year as refugees or asylum seekers. Countries were categorised as low-, middle- (LMIC) and in high-income (HIC) based on the World Bank list of economies 2020 (WBC, 2020).

### Quality assessment

Two reviewers (MP/MS or SG) evaluated the assessment of the methodological quality of individual studies using a modified version of the Joanna Briggs tool – JBI Critical Appraisal Checklist for Studies Reporting Prevalence Data (Munn *et al*., 2015) based on five questions, which permitted a critical assessment of prevalence rates: (1) Were study participants recruited in an appropriate way? (2) Was the sample size adequate? (3) Were the study subjects and the setting described in detail? (4) Was the condition measured in a standard, reliable way for all participants? (5) Was the response rate adequate, and if not, was the low response rate managed appropriately?. The response categories per each question were yes, unclear, and no. Three or more unclear or negative answers were considered to define a study with a high risk of bias (see online Supplementary materials). Any disagreements were resolved through discussion or by involving a third reviewer (PC).

### Data synthesis and statistical analysis

The prevalence rates of serious mental disorders were calculated by pooling the study-specific estimates. To stabilise the variance of binomial data, the Freeman-Tukey double arcsine transformation was used (Miller, 1978). Because we expected considerable heterogeneity between studies, we computed the pooled estimates under the random effects (DerSimonian and Laird) model based on the transformed values and their variance (Nyaga *et al*., 2014). The *I*^2^ was calculated as an indicator of the inter-study heterogeneity in percentages. Values of *I*^2^ are considered low, moderate and high when *I*^2^ equals 25, 50, and 75% respectively (Melsen *et al*., 2014). Based on previous meta-analyses on prevalence rates, we expected considerable heterogeneity in the prevalence estimates (Higgins, 2008; Charlson *et al*., 2019; Blackmore *et al*., 2020). To examine possible sources of heterogeneity between the included studies, we ran subgroups under the mixed-effects model with inverse-variance weights under the random-effects model (time since displacement ⩾ 5 years *v.* time since displacement >1 < 5years *v.* time since displacement <1year, MINI *v.* other diagnostic interviews, LMICs *v.* HICs, high risk of bias *v.* low risk of bias, convenience samples *v.* probability-based samples). Prevalence rates were calculated per each disorder separately. Pooled rates for subgroups were indicated, when at least three studies were present for each disorder. All statistical analyses were conducted using Meta-prop package in STATA/SE 16.1 for Mac (Nyaga *et al*., 2014).

## Results

**Fig. 1.**
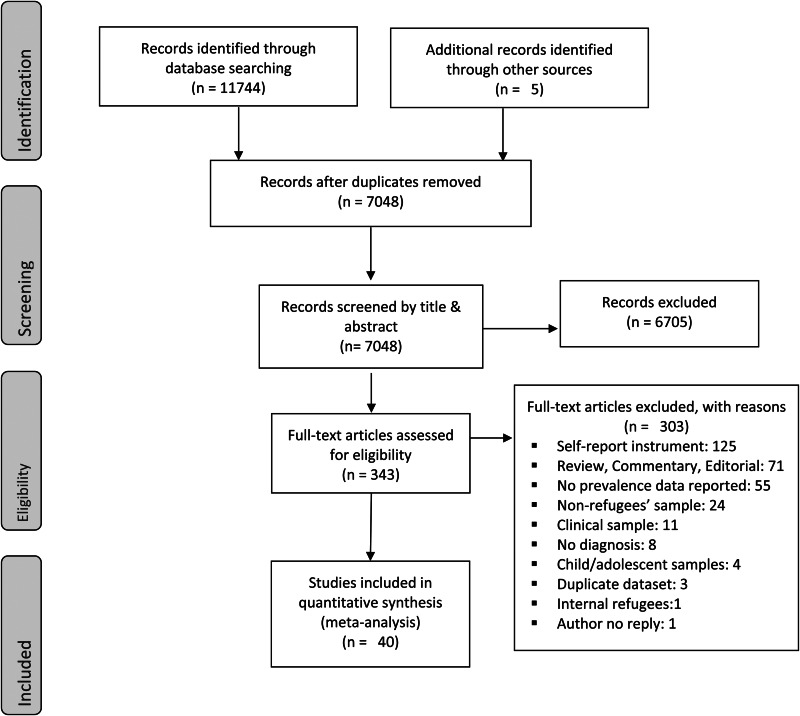
Study identification, screening, and eligibility test, following the Preferred Reporting Items of Systematic Reviews (PRISMA).

We initially identified 11 749 records. After exclusion of duplicates, 7048 records remained. We excluded 6705 studies based on titles and abstracts selection. We examined 343 full texts against our eligibility criteria and included 40 studies in the present meta-analysis (Fig. 1).

Studies included were conducted across 18 countries, 7 LMICs (Van Ommeren *et al*., 2001*a*; Akinyemi *et al*., 2012; Llosa *et al*., 2014; Naja *et al*., 2016; Tekin *et al*., 2016; Kazour *et al*., 2017; Segal *et al*., 2018; Tekeli-Yesil *et al*., 2018; Ainamani *et al*., 2020; Bapolisi *et al*., 2020; Civan Kahve *et al*., 2020; Kaur *et al*., 2020; Sagaltici *et al*., 2020) (Lebanon, Malaysia, Nepal, Nigeria, Turkey, Syria and Uganda) and 11 HICs (Hinton *et al*., 1993; Cheung, 1994; Steel *et al*., 2002; Turner *et al*., 2003; Fenta *et al*., 2004; Laban *et al*., 2004; Momartin *et al*., 2004; Marshall *et al*., 2005; Bhui *et al*., 2006; Renner *et al*., 2006; Von Lersner *et al*., 2008; Maier *et al*., 2010; Eckart *et al*., 2011; Jakobsen *et al*., 2011; Bogic *et al*., 2012; Heeren *et al*., 2012; Rasmussen *et al*., 2012; Tay *et al*., 2013; Hocking *et al*., 2015; 2018; Wright *et al*., 2017; Kizilhan, 2018; Nose *et al*., 2018; Richter *et al*., 2018; Rees *et al*., 2019; Wulfes *et al*., 2019; Sundvall *et al*., 2020) (Australia, Austria, Canada, Germany, Italy, New Zealand, Norway, Switzerland, The Netherlands, United Kingdom and United States of America) and they reported the prevalence of mental disorders of 11 053 participants (5309 men – 48%). Of these 11 053 participants, 6897 (62.3%) had taken part in population-based representative surveys (PBR). The majority of the studies included have used MINI as diagnostic interview (Bhui *et al*., 2006; Von Lersner *et al*., 2008; Maier *et al*., 2010; Akinyemi *et al*., 2012; Bogic *et al*., 2012; Heeren *et al*., 2012; Llosa *et al*., 2014; Hocking *et al*., 2015, 2018; Naja *et al*., 2016; Kazour *et al*., 2017; Nose *et al*., 2018; Richter *et al*., 2018; Segal *et al*., 2018; Tekeli-Yesil *et al*., 2018; Rees *et al*., 2019; Bapolisi *et al*., 2020; Kaur *et al*., 2020; Sundvall *et al*., 2020). CIDI was used by 7 studies (Van Ommeren *et al*., 2001*a*; Steel *et al*., 2002; Fenta *et al*., 2004; Laban *et al*., 2004; Marshall *et al*., 2005; Jakobsen *et al*., 2011; Rasmussen *et al*., 2012), SCID by six studies (Hinton *et al*., 1993; Tay *et al*., 2013; Tekin *et al*., 2016; Wright *et al*., 2017; Kizilhan, 2018; Wulfes *et al*., 2019) and one study (Cheung, 1994) used the Diagnostic Interview Schedule – DIS. The Clinician-Administered PTSD Scale – CAPS and the PTSD Symptom Scale–Interview- PSS-I were used by six studies (Turner *et al*., 2003; Momartin *et al*., 2004; Renner *et al*., 2006; Eckart *et al*., 2011; Civan Kahve *et al*., 2020; Sagaltici *et al*., 2020) and one study (Ainamani *et al*., 2020) respectively (Table 1).

**Table 1.** Studies meeting inclusion criteria

Study	Country of study	Country category	Population	Sample size, *n*	*PBR*	Age, *n* – M (s.d.)	Gender, *n*	Time since displacement (years)	Questionnaire	MDD, *n*	MDD Recurrent ep, *n*	PTSD,*n*	Bipolar disorders, *n*	Pychotic disorders, *n* (%)	RoB
Ainamani *et al*. (2020)	Uganda	LMIC	Congolese	325	No	M 31.9 F 30.9	M 143 F 182	NR	PSS-I	NR	NR	285/325	NR	NR	High
Akinyemi *et al*. (2012)	Nigeria	LMIC	Liberian, Sierra Leonean, Togolese	444	Yes	34.8 (±12.8)	M 263 F 181	⩾5	MINI	201/444	NR	150/444	114/444	NR	Low
Bapolisi *et al*. (2020)	Uganda	LMIC	Congolese, Burundian, Somali, Rwandese, Ethiopian, Eritrean, Sudanese	387	Yes	33.01 (±12.2)	M 168 F 219	>1 < 5	MINI	226/387	NR	260/387	NR	NR	Low
Bhui *et al*. (2006)	UK	HIC	Somali	143	No	⩾18	M 71 F 72	<1	MINI	38/143	13/143	20/143	0/143	1/143	Low
Bogic *et al*. (2012)	Germany, Italy and UK	HIC	Former Yugoslavia	854	Yes	41.6 (±10.8)	M 416 F 438	⩾5	MINI	292/851	132/846	282/854	16/850	11/854	High
Cheung (1994)	New Zealand	HIC	Cambodian	223	Yes	⩾18	M 104 F 119	>1 < 5	DIS	NR	NR	27/223	NR	NR	Low
Civan Kahve *et al*. (2020)	Turkey	LMIC	Iraqi	101	No		M 49 F 52	>1 < 5	CAPS	NR	NR	9/101	NR	NR	Low
Eckart *et al*. (2011)	Germany	HIC	Albanian, Kurdish, Romanian, Serbian, Turkish	52	No	⩾18	M 52 F 0	NR	CAPS MINI	17/52	NR	20/52	NR	NR	High
Fenta *et al*. (2004)	Canada	HIC	Ethiopian	325	No	35.3 (±7.2)	M 203 F 139	⩾5	CIDI	20/325	33/342	NR	NR	NR	High
Heeren *et al*. (2012)	Switzerland	HIC	African, Asian, European	86	No	NR	M 60 F 26	NR	MINI	27/86	NR	20/86	NR	NR	High
Hinton *et al*. (1993)	USA	HIC	Vietnamese, Chinese	201	NR	33.2 (±12.0)	M 96 F 105	<1	SCID	11/201	NR	7/201	NR	NR	High
Hocking *et al*. (2015)	Australia	HIC	Sri Lanka, Pakistan, Zimbabwe, Iraqi, Afghan, Iranian, Lebanon	98	No	34.6 (±10.6)	M 87 F 11	NR	MINI	58/95	NR	49/94	NR	NR	High
Hocking *et al*. (2018)	Australia	HIC	Asia	185	NR	⩾18	M 129 F 56	>1 < 5	MINI	56/185	NR	NR	NR	NR	High
Jakobsen *et al*. (2011)	Norway	HIC	Middle East, North African, Somali, Former Yugoslavia	64	No	33 (±11.6)	M 34 F 30	>1 < 5	CIDI	21/64	NR	29/64	NR	NR	Low
Kaur *et al*. (2020)	Malaysia	LMIC	Rohingya	220	Yes	33.5	M 116 F 104	>1 < 5	MINI	71/220	NR	84/220	NR	NR	Low
Kazour *et al*. (2017)	Lebanon	LMIC	Syrian	452	No	35.05 (±12.3)	M 200 F 252	<1	MINI	NR	NR	123/452	NR	NR	High
Kizilhan (2018)	Germany	HIC	Yazidi	296	No	23.72 (±2.62)	F 296	>1 < 5	SCID	158/296	NR	144/296	NR	NR	High
Laban *et al*. (2004)	The Netherlands	HIC	Iraqi	294	Yes	⩾18	M 190 F 104	>1 < 5	CIDI	85/294	13/294	108/294	NR	NR	Low
Llosa *et al*. (2014)	Lebanon	LMIC	Palestinian	194	Yes	41.5 (±15.1)	M 56 F 138	NR	MINI	31/194	48/194	9/194	3/194	5/194	Low
Maier *et al*. (2010)	Switzerland	HIC	Asian, African, European	78	No	29.9 (± 8.4)	M 57 F 21	>1 < 5	MINI	26/78	NR	19/78	NR	NR	High
Marshall *et al*. (2005)	USA	HIC	Cambodian	490	Yes	52 (±13.4)	M 171 F 319	⩾5	CIDI	248/490	NR	301/490	NR	NR	Low
Momartin *et al*. (2004)	Australia	HIC	Bosnian	126	No	47	M 49 F 77	NR	CAPS	NR	NR	79/126	NR	NR	Low
Naja *et al*. (2016)	Lebanon	LMIC	Syrian	310	No	18-65	M 120 F 189	NR	MINI	136/310	NR	NR	NR	NR	High
Nose *et al*. (2018)	Italy	HIC	Pakistan, Afghan, African, Ex-Yugoslavia	109	No	30.0 (±7.60)	M 109	>1 < 5	MINI	6/109	NR	9/109	NR	NR	Low
Rasmussen *et al*. (2012)	USA	HIC	Mexican, South America, Chinese, Vietnamese	660	Yes	⩾18	M 345 F 315	NR	CIDI	97/660	NR	8/660	NR	NR	Low
Rees *et al*. (2019)	Australia	HIC	Arabic countries, Sri Lanka, Sudan.	289	Yes	30.0 (±75.8)	F 289	<1 >1 < 5 ⩾5	MINI	94/289	NR	NR	NR	NR	Low
Renner *et al*. (2006)	Austria	HIC	Chechnya, Afghan, West African	150	No	⩾18	M 110 F 40	NR	CAPS	NR	NR	32/150	NR	NR	High
Richter *et al*. (2018)	Germany	HIC	Iraninan, Russian, Iraqi, Afghan, Azerbaijan	125	No	31.9 (± 0.6)	M 83 F 42	<1	MINI	9/125	7/125	22/125	NR	1/125	High
Sagaltici *et al*. (2020)	Turkey	LMIC	Syrian	342	Yes	33.7 (±10.0)	M 163 F 179	>1 < 5	CAPS	NR	NR	106/342	NR	NR	High
Segal *et al*. (2018)	Lebanon	LMIC	Syrian, Palestinian	254	No	40.4 (±13.5)	M 114 F 140	>1 < 5	Modified-MINI	NR	NR	13/254	NR	2/254	High
Steel *et al*. (2002)	Australia	HIC	Vietnamese	1161	Yes	41 (±14.2)	M 472 F 689	⩾5	CIDI	29/1161	NR	51/1161	NR	NR	Low
Sundvall *et al*. (2020)	Sweden	HIC	Iraqi	31	No	48	F 14 M 17	⩾5	MINI	9/30	NR	13/30	NR	NR	High
Tay *et al*. (2013)	Australia	HIC	Afghan, Chinese, Ganese, Iranian, Zimbabwe	52	No	39 (±13.5)	M 34 F18	<1	SCID	30/52	NR	31/52	NR	NR	Low
Tekeli-Yesil *et al*. (2018)	Turkey – Syria	LMIC	Syrian	285	No	34.19 (± 1.7)	M 144 F 141	>1 < 5	MINI	201/285	104/238	85/285	NR	NR	High
Tekin *et al*. (2016)	Turkey	LMIC	Iraqi	238	Yes	32.70 (±11.8)	M 105 F 133	<1	SCID	94/238	NR	102/238	NR	NR	Low
Turner *et al*. (2003)	UK	HIC	Kosovo	120	No	37.1	M 56 F 64	NR	CAPS	NR	NR	46/118	NR	NR	High
Van Ommeren *et al*. (2001*a*, 2001*b*)	Nepal	LMIC	Bhutanese	810	Yes	⩾18	M 616 F 194	⩾5	CIDI	23/810	139/810	197/810	NR	NR	Low
von Lersner *et al*. (2008)	Germany	HIC	former Yugoslavia, Turkhis	53	No	38.3 (±10.3)	M 25 F 28	⩾5	MINI	27/53	NR	29/53	1/53	NR	Low
Wright *et al*. (2017)	USA	HIC	Iraqi	291	Yes	34.30 (±11.3)	M 158 F 133	NR	SCID	8/291	NR	11/291	NR	NR	Low
Wulfes *et al*. (2019)	Germany	HIC	Afghan, Iranian, Iraqi, Syrian, Sudan	118	No	32.9 (±13.1)	M 76 F 42	NR	SCID	39/118	NR	35/118	NR	NR	High

PBR, population-based representative surveys; CAPS, Clinician-Administered PTSD Scale; CIDI, Composite International Diagnostic Interview; DIS, Diagnostic Interview Schedule; HIC, High-income country; LMIC, Low middle-income country; MDD, Major depressive disorder; MINI, The Mini-International Neuropsychiatric Interview; NR, Not reported; PSS-I, PTSD Symptom Scale–Interview; SCID, The Structured Clinical Interview for DS.

The prevalence of MDD was reported in 31 studies (Hinton *et al*., 1993; Van Ommeren *et al*., 2001*a*; Steel *et al*., 2002; Fenta *et al*., 2004; Laban *et al*., 2004; Marshall *et al*., 2005; Bhui *et al*., 2006; Von Lersner *et al*., 2008; Maier *et al*., 2010; Eckart *et al*., 2011; Jakobsen *et al*., 2011; Akinyemi *et al*., 2012; Bogic *et al*., 2012; Heeren *et al*., 2012; Rasmussen *et al*., 2012; Tay *et al*., 2013; Llosa *et al*., 2014; Hocking *et al*., 2015, 2018; Naja *et al*., 2016; Tekin *et al*., 2016; Wright *et al*., 2017; Kizilhan, 2018; Nose *et al*., 2018; Richter *et al*., 2018; Tekeli-Yesil *et al*., 2018; Rees *et al*., 2019; Wulfes *et al*., 2019; Bapolisi *et al*., 2020; Kaur *et al*., 2020; Sundvall *et al*., 2020): 23 reported only the prevalence of a current episode of MDD (Hinton *et al*., 1993; Steel *et al*., 2002; Marshall *et al*., 2005; Von Lersner *et al*., 2008; Maier *et al*., 2010; Eckart *et al*., 2011; Jakobsen *et al*., 2011; Akinyemi *et al*., 2012; Heeren *et al*., 2012; Rasmussen *et al*., 2012; Tay *et al*., 2013; Hocking *et al*., 2015, 2018; Naja *et al*., 2016; Tekin *et al*., 2016; Wright *et al*., 2017; Kizilhan, 2018; Nose *et al*., 2018; Rees *et al*., 2019; Wulfes *et al*., 2019; Bapolisi *et al*., 2020; Kaur *et al*., 2020; Sundvall *et al*., 2020) (MDD) and 8 reported also prevalence of recurrent episode of MDD (reMDD) (Van Ommeren *et al*., 2001*a*; Fenta *et al*., 2004; Laban *et al*., 2004; Bhui *et al*., 2006; Bogic *et al*., 2012; Llosa *et al*., 2014; Richter *et al*., 2018; Tekeli-Yesil *et al*., 2018) (Table 1). The pooled prevalence rate of MDD was 32% (95% CI 26–39%) with a very high heterogeneity between sample (*I*^2^ = 99.05%; *p* = 0.000) (Table 2 and figure 2.1). The random-effects pooled prevalence of reMDD was 16% (95% CI 10–22%) and the rates ranged from 4% to 44% (*I*^2^ = 96.3%; *p* = 0.000) (Table 2 and figure 2.2).

**Table 2.** Prevalence rates of serious mental disorders

Mental disorder	Prevalence rate (%)	Prevalence rates	Heterogeneity (*I*^2^) (%)	*p*
		Random pooled ES (95% CI)		
MDD	32	26–39%	99.05	0⋅000
MDD (outliers removed)	33	31–35%	0.0	0.64
MDD recurrent episode	16	10–22%	96.3	0⋅000
PTSD	31	25–38%	99.3	0⋅000
PTSD (outliers removed)	32	30–35%	50.2	0.05
BPD	5	2–9%	97.7	0⋅000
Psychosis	1	1–2%	0.00	0⋅000

MDD, Major depressive disorder; PTSD, Post-traumatic stress disorder; BPD, Bipolar disorder.

The prevalence of PTSD was reported in 36 studies (Hinton *et al*., 1993; Cheung, 1994; Van Ommeren *et al*., 2001*b*; Steel *et al*., 2002; Turner *et al*., 2003; Laban *et al*., 2004; Momartin *et al*., 2004; Marshall *et al*., 2005; Bhui *et al*., 2006; Renner *et al*., 2006; Von Lersner *et al*., 2008; Maier *et al*., 2010; Eckart *et al*., 2011; Jakobsen *et al*., 2011; Akinyemi *et al*., 2012; Bogic *et al*., 2012; Heeren *et al*., 2012; Rasmussen *et al*., 2012; Tay *et al*., 2013; Llosa *et al*., 2014; Hocking *et al*., 2015; Tekin *et al*., 2016; Kazour *et al*., 2017; Wright *et al*., 2017; Kizilhan, 2018; Nose *et al*., 2018; Richter *et al*., 2018; Segal *et al*., 2018; Tekeli-Yesil *et al*., 2018; Wulfes *et al*., 2019; Ainamani *et al*., 2020; Bapolisi *et al*., 2020; Civan Kahve *et al*., 2020; Kaur *et al*., 2020; Sagaltici *et al*., 2020; Sundvall *et al*., 2020) (Table 1). The random-effects pooled prevalence of PTSD was 31% (95% CI 25–38%) and the rates ranged from 1% to 88%. The heterogeneity between samples was high (*I*^2^ = 99.3%; *p* = 0.000) (Table 2 and figure 2.3).

The prevalence of BPD was reported in five articles (Bhui *et al*., 2006; Von Lersner *et al*., 2008; Akinyemi *et al*., 2012; Bogic *et al*., 2012; Llosa *et al*., 2014) (Table 1) and the random-effects pooled prevalence was 5% (95% CI 2–9%; *I*^2^ = 97.7%) (Table 2 and figure 2.4).

The prevalence of psychotic disorders was reported in five articles (Bhui *et al*., 2006; Bogic *et al*., 2012; Llosa *et al*., 2014; Richter *et al*., 2018; Segal *et al*., 2018) (Table 1). The random-effects pooled prevalence of the psychosis was 1% (95% CI 1–2%; *I^2^* = 0.00%) (Table 2 and figure 2.5).

To reduce the high heterogeneity and confirm our prevalence rates, we also ran analyses without the outliers when it was possible. The analysis showed a prevalence of 33% of MDD and 32% of PTSD (Table 2 and online Supplementary materials).

Subgroups analysis showed a significant prevalence rates difference (*p* = 0.001) of MDD between studies conducted in refugees resettled in LMICs (Akinyemi *et al*., 2012; Llosa *et al*., 2014; Naja *et al*., 2016; Tekin *et al*., 2016; Tekeli-Yesil *et al*., 2018; Bapolisi *et al*., 2020; Kaur *et al*., 2020) (47%; 95% CI 38–57%) and those in HICs (Hinton *et al*., 1993; Van Ommeren *et al*., 2001*a*; Steel *et al*., 2002; Fenta *et al*., 2004; Laban *et al*., 2004; Momartin *et al*., 2004; Marshall *et al*., 2005; Bhui *et al*., 2006; Von Lersner *et al*., 2008; Maier *et al*., 2010; Eckart *et al*., 2011; Jakobsen *et al*., 2011; Bogic *et al*., 2012; Heeren *et al*., 2012; Rasmussen *et al*., 2012; Tay *et al*., 2013; Hocking *et al*., 2015, 2018; Wright *et al*., 2017; Kizilhan, 2018; Nose *et al*., 2018; Richter *et al*., 2018; Rees *et al*., 2019; Wulfes *et al*., 2019; Sundvall *et al*., 2020) (28%; 95% CI 22–33%) (Table 3 and figure 3.1). Besides, a significant (*p* = 0.05) higher prevalence rate of MDD (37%; 95% CI 28–46%) has been reported in studies (Bhui *et al*., 2006; Von Lersner *et al*., 2008; Maier *et al*., 2010; Eckart *et al*., 2011; Akinyemi *et al*., 2012; Bogic *et al*., 2012; Heeren *et al*., 2012; Llosa *et al*., 2014; Hocking *et al*., 2015, 2018; Naja *et al*., 2016; Nose *et al*., 2018; Richter *et al*., 2018; Tekeli-Yesil *et al*., 2018; Rees *et al*., 2019; Bapolisi *et al*., 2020; Kaur *et al*., 2020; Sundvall *et al*., 2020) conducted using the MINI as compared to studies that used others diagnostic interviews (Hinton *et al*., 1993; Van Ommeren *et al*., 2001*a*; Steel *et al*., 2002; Fenta *et al*., 2004; Laban *et al*., 2004; Momartin *et al*., 2004; Marshall *et al*., 2005; Jakobsen *et al*., 2011; Rasmussen *et al*., 2012; Tay *et al*., 2013; Tekin *et al*., 2016; Wright *et al*., 2017; Kizilhan, 2018; Wulfes *et al*., 2019) (26%; 95% CI 20–33%) (Table 3 and figure 3.2). In addition, studies (Turner *et al*., 2003; Fenta *et al*., 2004; Momartin *et al*., 2004; Bhui *et al*., 2006; Renner *et al*., 2006; Von Lersner *et al*., 2008; Maier *et al*., 2010; Eckart *et al*., 2011; Jakobsen *et al*., 2011; Heeren *et al*., 2012; Tay *et al*., 2013; Naja *et al*., 2016; Kazour *et al*., 2017; Hocking *et al*., 2018; Kizilhan, 2018; Nose *et al*., 2018; Richter *et al*., 2018; Segal *et al*., 2018; Tekeli-Yesil *et al*., 2018; Wulfes *et al*., 2019; Ainamani *et al*., 2020; Civan Kahve *et al*., 2020; Sundvall *et al*., 2020) which included participants from a convenience samples showed significant (*p* = 0.001) higher prevalence of MDD and PTSD (MDD: 35%; 95% CI 23–46%; PTSD: 34%; 95% CI 22–47%) than studies (Cheung, 1994; Van Ommeren *et al*., 2001*a*; Steel *et al*., 2002; Laban *et al*., 2004; Marshall *et al*., 2005; Akinyemi *et al*., 2012; Bogic *et al*., 2012; Rasmussen *et al*., 2012; Llosa *et al*., 2014; Tekin *et al*., 2016; Wright *et al*., 2017; Rees *et al*., 2019; Bapolisi *et al*., 2020; Kaur *et al*., 2020; Sagaltici *et al*., 2020) conducted among probability-based samples (MDD: 30%; 95% CI 21–39%; PTSD: 28% 95% CI 19–37%) (Table 3 and figure 3.3). The heterogeneity between samples was high in all the subgroup analyses (*I*^2^ = 99.05%). The other analyses of the MDD, reMDD and PTSD subgroups, showed no significant differences among prevalence rates and high heterogeneity (Table 3 and online Supplementary materials). For psychotic disorders and BPD subgroup analyses were not conducted due to the limited number of studies.

**Table 3:** Subgroups analysis results

Major Depression Disorder (MDD)			
Subgroup	Categories	Random pooled ES (95% CI)	*p*
Time since displacement	(⩾5)	27 (16–37)	0.55
	(>1 < 5)	36 (20–51)	
	(<1)	26 (11–42)	
Diagnostic interviews	MINI	37 (28–46)	**0.05**
	Others*	26 (20–33)	
Country categories	LMICs	47 (38–57)	**0.001**
	HICs	28 (22–33)	
Risk of bias	Low	31 (24–39)	0.74
	High	34 (22–45)	
Population	Probability-based samples	30 (21–39)	**0.001**
	Convenience samples	35 (23–46)	
Recurrent episode of MDD (reMDD)				
Time since displacement	(⩾5)	NP	NP
	(>1 < 5)		
	(<1)		
Diagnostic interviews	MINI	20 (9–30)	0.168
	Others*	10 (3–8)	
Country categories	LMICs	NP	NP
	HICs		
Risk of bias	Low	14 (5–22)	0.507
	High	18 (7–29)	
Population	Probability-based samples	15 (8–23)	0.834
	Convenience samples	17 (4–30)	
Post-traumatic stress disorder				
Time since displacement	(⩾5)	35 (17–53)	0.908
	(>1 < 5)	31 (20–42)	
	(<1)	27 (13–41)	
Diagnostic interviews	MINI	30 (21–39)	0.961
	Others*	33 (23–42)	
Country categories	LMICs	34 (17–52)	0.641
	HICs	30 (23–36)	
Risk of bias	Low	30 (23–38)	0.757
	High	33 (19–46)	
Population	Probability-based samples	28 (19–37)	**0.001**
	Convenience samples	34 (22–47)	

* Others: (CAPS, Clinician-Administered PTSD Scale; CIDI, Composite International Diagnostic Interview; DIS, Diagnostic Interview Schedule; SCID, The Structured Clinical Interview for DSM); MINI, The Mini-International Neuropsychiatric Interview; (⩾5): people who have spent more than five years as refugees or asylum seekers; (>1 < 5): people who have spent between 5 years and 1 year as refugees or asylum seekers; (<1): people who have spent less than 1 year as refugees or asylum seekers; HICs, High-income countries; LMICs, Low middle-income countries; NP, The aggregate prevalence of random effects was not allowed due to the limited number of the sample.

## Discussion

This systematic review and meta-analysis provide the most recent and extensive overview of the prevalence rates of serious mental disorders across refugees and asylum-seeking populations. A comprehensive search was performed from inception to June 4, 2020 and was not intentionally updated to avoid interference caused by the effect of COVID-19 pandemic in this population. We included 40 studies in 11 053 participants, and all diagnoses were established with diagnostic interviews. Our study revealed that the most prevalent serious mental disorder in refugees and asylum seekers was MDD (32%), followed by PTSD (31%), recurrent episode of MDD (16%), and BPD (5%). The prevalence of psychotic disorders was 1%. Subgroup analyses showed that MDD appeared to be more prevalent (47%) among studies conducted in LMICs than in HICs (28%), and when the MINI (37%) has been used, compared to other diagnostic instruments (26%). PTSD and MDD showed higher prevalence rates (34% and 35% respectively) in studies where participants were from convenience samples in comparison to studies that used probability-based samples. PTSD and MDD were the most frequently evaluated disorders with 36 studies and 32 studies respectively, as opposed to BPD and psychosis of which we had only a few studies.

According to previous studies, the world-wide prevalence rate reported for MDD is 4.4% (WHO, 2017), the lifetime prevalence of PTSD in World Mental Health Surveys has been calculated between 3.9% and 5.6% (Koenen *et al*., 2017) and the prevalence rates of psychosis and BPD in the general population are 0.4% (Moreno-Kustner *et al*., 2018) and 2.5%, respectively (Carta and Angst, 2016). Comparing our results with the prevalence of the same disorders in the general population, suggests that MDD is seven times more likely in refugees and PTSD is 4 to 5 times more prevalent than in the general population (Koenen *et al*., 2017; WHO, 2017). Although BPD and psychosis are much rarer than MDD or PTSD, our study also indicated that externally displaced refugees and asylum seekers are two times more likely to be diagnosed with BPD and with psychosis than the general population (Carta and Angst, 2016; Moreno-Kustner *et al*., 2018). Studies have highlighted the role of trauma and stress experienced by minorities, social defeat, and discrimination as important risk factors for psychosis in refugees (Brandt *et al*., 2019; Duggal *et al*., 2020; Selten *et al*., 2020).

Our results are in line with the prevalence rates of the Blackmore and colleagues (Blackmore *et al*., 2020) study, who found a prevalence rates of 31.4% (95% CI 24.43–38.5) for PTSD, of 31.5% (95% CI 22.64–40.38) for depression, and 1.5% (95% CI 0.63–2.40) for psychosis. In addition, our study reports the random-effects pooled prevalence for BPD which has never examined before. Compared to the 2005 systematic review findings (Fazel *et al*., 2005), our rates prevalence showed an increase of serious mental disorders prevalence among refugees and asylum seekers from 5% (Fazel *et al*., 2005) to 32% for MDD, and from 9% (Fazel *et al*., 2005) to 31% for PTSD. One of the reasons for the discrepancy between these results could be the LMICs exclusion in Fazel and colleagues' systematic review. Furthermore, it might be explained by increased exposure to adverse events, increased financial hardship, social isolation, decreased access to adequate health care, as well as the lack of appropriate policies and investments in the last 15 years.

Our study methodology has several strengths, among which strict inclusion and exclusion criteria, a large number of included studies, and updated statistical methods. However, due to the low number of studies that examine refugees and asylum seekers separately, a limitation of this study was to consider these two groups together. Further, we found a large variability of prevalence rates and a high level of heterogeneity between studies. While high heterogeneity is very common in meta-analyses on prevalence rates (Higgins, 2008), we examined possible sources of heterogeneity between the included studies. Running the analyses without outliers did not alter the main results. Upon inspection of the pattern of outliers, no systematic differences between outlier studies from the rest of the studies were identified that might explain the high heterogeneity. However, due to the limit number of studies greater caution is required in interpreting the prevalence rates of BP and psychosis. In addition, we analysed subgroups under the mixed-effects model with inverse-variance weights under the random-effects model. The results of these subgroup analyses showed that significantly higher prevalence rates of MDD and PTSD in studies conducted among convenience samples than in studies that used probability-based samples. This result highlights the importance of having an adequate statistically representative sample of participants to avoid overestimating prevalence rates in a population as various and difficult to study as that of refugees and asylum seekers.

Furthermore, higher prevalence rates of MDD were found in LMICs than in HICs. This difference was not found for PTSD, where the prevalence rate was comparable between refugees and asylum seekers resettled in LMICs and HICs. The higher prevalence of MDD in refugees resettled in LMICs may be driven by higher exposure to post-migration living difficulties. Feelings of hopelessness, the failure of the migration project, and difficulties in integration may promote higher levels of depression (Steel *et al*., 2009; Charlson *et al*., 2019). Further, refugees resettled in LMICs can have a higher risk of developing MDD because of the lack of integration programs and mental health care due to the low investments in mental health care that unfortunately characterises the majority of these countries (Patel, 2007). On the other hand, compared to MDD, PTSD may have a stronger association with trauma exposure in the country of origin, which may not differ significantly between refugees in LMICs and HICs.

Another interesting finding was that we found a higher rate for MDD for studies conducted with the MINI than in studies that used different diagnostic interviews. This may be explained by the fact that the MINI may be administered by non-clinicians (similar to the CIDI, but dissimilar to the SCID I/P), and that its administration is shorter and its outcomes therefore potentially less precise.

Despite the high heterogeneity and methodological limitations of included studies, a high prevalence of serious mental disorders was found in refugees and asylum seekers diagnosed through structured clinical interviews. However, specific instruments culturally adapted for use across different local cultures and contexts for refugees would be advisable, since no one of the diagnostic instruments currently in use has been developed for the non-western populations, who represent the highest sample of refugees. For this reason, to reduce the high heterogeneity, more rigorous studies, using representative samples and culturally adapted instruments, to measure cultural concepts of distress would be recommended. Moreover, to strengthen the evidence base concerning the more serious mental disorders, further research on the prevalence of psychosis and BPD is imperative. To follow this goal, international and government investments in mental health research, population screening, and specific interventions on asylum seekers and refugees are warranted. However, until we have more rigorous studies and adequate diagnostic tools, due to the high heterogeneity between these studies, our results should be considered with caution.

## Conclusion

In sum, our systematic review and meta-analysis show that it is imperative that governments and actors across the world acknowledge the devastating effect of war and prosecution on individuals' mental health. In order to prevent cycles of violence and victimisation, effective public mental health responses may be put in place to address worldwide suffering.

## Data

All the data involved have been included in Tables and Figures of this paper, including online Supplementary materials.

## Supplementary material

For supplementary material accompanying this paper visit https://doi.org/10.1017/gmh.2022.29. click here to view supplementary material
