# Supplementary material for: Prevalence of mental disorders in refugees and asylum seekers: a systematic review and meta-analysis
Source: Glob Ment Health (Camb). 2022 Jun 14;9:250–63. doi: 10.1017/gmh.2022.29 (PMC9806970; doi:10.1017/gmh.2022.29)
Supplement: Supplementary file 1 [file S2054425122000292sup001.docx]

**Supplementary material**

Contents

- PRISMA Checklist 2
- [Electronic databases searched 5](#_Toc359837898)
- [Search terms for Pubmed, Embase, APA PsycInfo 5](#_Toc359837899)
- [Quality Appraisal Checklist 8](#_Toc359837901)
- Quality of included studies………………………………………………….……..10
- Supplementary forest plots: MDD and PTSD without outliers and subgroups (by disorders)...12

**PRISMA Checklist**

| **Section/topic** | **#** | **Checklist item** | **Reported on page #** |
| --- | --- | --- | --- |
| **TITLE** | | |  |
| Title | 1 | Identify the report as a systematic review, meta-analysis, or both. | Title |
| **ABSTRACT** | | |  |
| Structured summary | 2 | Provide a structured summary including, as applicable: background; objectives; data sources; study eligibility criteria, participants, and interventions; study appraisal and synthesis methods; results; limitations; conclusions and implications of key findings; systematic review registration number. | Abstract |
| **INTRODUCTION** | | |  |
| Rationale | 3 | Describe the rationale for the review in the context of what is already known. | 4 and 5 |
| Objectives | 4 | Provide an explicit statement of questions being addressed with reference to participants, interventions, comparisons, outcomes, and study design (PICOS). | 6 |
| **METHODS** | | |  |
| Protocol and registration | 5 | Indicate if a review protocol exists, if and where it can be accessed (e.g., Web address), and, if available, provide registration information including registration number. | 7 |
| Eligibility criteria | 6 | Specify study characteristics (e.g., PICOS, length of follow-up) and report characteristics (e.g., years considered, language, publication status) used as criteria for eligibility, giving rationale. | 7 and 8 |
| Information sources | 7 | Describe all information sources (e.g., databases with dates of coverage, contact with study authors to identify additional studies) in the search and date last searched. | 7 and 8 |
| Search | 8 | Present full electronic search strategy for at least one database, including any limits used, such that it could be repeated. | Supplementary materials |
| Study selection | 9 | State the process for selecting studies (i.e., screening, eligibility, included in systematic review, and, if applicable, included in the meta-analysis). | 8-10 |
| Data collection process | 10 | Describe method of data extraction from reports (e.g., piloted forms, independently, in duplicate) and any processes for obtaining and confirming data from investigators. | 8-10 |
| Data items | 11 | List and define all variables for which data were sought (e.g., PICOS, funding sources) and any assumptions and simplifications made. | 8-10 |
| Risk of bias in individual studies | 12 | Describe methods used for assessing risk of bias of individual studies (including specification of whether this was done at the study or outcome level), and how this information is to be used in any data synthesis. | 9 and Supplementary materials |
| Summary measures | 13 | State the principal summary measures (e.g., risk ratio, difference in means). | 9 and 10 |
| Synthesis of results | 14 | Describe the methods of handling data and combining results of studies, if done, including measures of consistency (e.g., I^2^) for each meta-analysis. | 9 and 10 |

| Risk of bias across studies | 15 | Specify any assessment of risk of bias that may affect the cumulative evidence (e.g., publication bias, selective reporting within studies). | 9 |
| --- | --- | --- | --- |
| Additional analyses | 16 | Describe methods of additional analyses (e.g., sensitivity or subgroup analyses, meta-regression), if done, indicating which were pre-specified. | 10 |
| **RESULTS** | | |  |
| Study selection | 17 | Give numbers of studies screened, assessed for eligibility, and included in the review, with reasons for exclusions at each stage, ideally with a flow diagram. | 10 |
| Study characteristics | 18 | For each study, present characteristics for which data were extracted (e.g., study size, PICOS, follow-up period) and provide the citations. | Table 1 |
| Risk of bias within studies | 19 | Present data on risk of bias of each study and, if available, any outcome level assessment (see item 12). | Supplementary materials and Table 3 |
| Results of individual studies | 20 | For all outcomes considered (benefits or harms), present, for each study: (a) simple summary data for each intervention group (b) effect estimates and confidence intervals, ideally with a forest plot. | Figures 2.1, 2.2, 2.3, 2.4, 2.5 and 3.1, 3.2 |
| Synthesis of results | 21 | Present results of each meta-analysis done, including confidence intervals and measures of consistency. | 10-13 and Table 2 |
| Risk of bias across studies | 22 | Present results of any assessment of risk of bias across studies (see Item 15). | Supplementary materials and Table 3 |
| Additional analysis | 23 | Give results of additional analyses, if done (e.g., sensitivity or subgroup analyses, meta-regression [see Item 16]). | 13 and 14 and Table 3 |
| **DISCUSSION** | | |  |
| Summary of evidence | 24 | Summarize the main findings including the strength of evidence for each main outcome; consider their relevance to key groups (e.g., healthcare providers, users, and policy makers). | 14 |
| Limitations | 25 | Discuss limitations at study and outcome level (e.g., risk of bias), and at review-level (e.g., incomplete retrieval of identified research, reporting bias). | 15 and 16 |
| Conclusions | 26 | Provide a general interpretation of the results in the context of other evidence, and implications for future research. | 17 |
| **FUNDING** | | |  |
| Funding | 27 | Describe sources of funding for the systematic review and other support (e.g., supply of data); role of funders for the systematic review. | 17 and 18 |

*From:*  Moher D, Liberati A, Tetzlaff J, Altman DG, The PRISMA Group (2009). Preferred Reporting Items for Systematic Reviews and Meta-Analyses: The PRISMA Statement. PLoS Med 6(7): e1000097. doi:10.1371/journal.pmed1000097 . For more information, visit: **www.prisma-statement.org**.

**Electronic database searched**

## Results February 4, 2021

| Database | Results |
| --- | --- |
| Pubmed | 3618 |
| Embase | 4520 |
| APA PsycInfo | 3606 |
| Results | 11744 |
| After removing duplicates | 7048 |

**Supplementary information S1**

### PubMed History February 4, 2021

| **Search** | **PubMed Query – February 4, 2021** | **Items found** |
| --- | --- | --- |
| #4 | #3 NOT (("Adolescent"[Mesh] OR "Child"[Mesh] OR "Infant"[Mesh] OR adolescen*[tiab] OR child*[tiab] OR schoolchild*[tiab] OR infant*[tiab] OR girl*[tiab] OR boy[tiab] OR boys[tiab] OR boyhood[tiab] OR teen[tiab] OR teens[tiab] OR teenager*[tiab] OR youth*[tiab] OR pediatr*[tiab] OR paediatr*[tiab] OR puber*[tiab]) NOT ("Adult"[Mesh] OR adult*[tiab] OR man[tiab] OR men[tiab] OR woman[tiab] OR women[tiab])) | 3,618 |
| #3 | #1 AND #2 | 4,369 |
| #2 | "Mental Disorders"[Mesh] OR "Suicide"[Mesh] OR “mental dis*”[tiab] OR “mental ill*”[tiab] OR “mentally ill*”[tiab] OR “mental health”[tiab] OR “affective disorder*”[tiab] OR “mood disorder*”[tiab] OR depress*[tiab] OR anxiety[tiab] OR bipolar[tiab] OR PTSD[tiab] OR psychosis[tiab] OR psychotic[tiab] OR schizophren*[tiab] OR “post-traumatic stress”[tiab] OR “posttraumatic stress”[tiab] OR mental[tiab] OR suicid*[tiab] | 1,932,762 |
| #1 | "Refugees"[Mesh] OR refugee*[tiab] OR “asylum seeker*”[tiab] OR “displaced person*”[tiab] OR “displaced people”[tiab] OR “displaced population*”[tiab] OR “displaced female”[tiab] OR “displaced male”[tiab] OR “displaced wom*”[tiab] OR “displaced men”[tiab] OR “displaced man”[tiab] OR “displaced individual*”[tiab] OR stateless*[tiab] | 15,739 |

### Embase.com History February 4, 2021

| **Search** | **Embase.com Query – February 4, 2021** | **Items found** |
| --- | --- | --- |
| #4 | #3 NOT (('adolescent'/exp OR 'child'/exp OR adolescent*:ti,ab OR child*:ti,ab OR schoolchild*:ti,ab OR infant*:ti,ab OR girl*:ti,ab OR boy*:ti,ab OR teen:ti,ab OR teens:ti,ab OR teenager*:ti,ab OR youth*:ti,ab OR pediatr*:ti,ab OR paediatr*:ti,ab OR puber*:ti,ab ) NOT ('adult'/exp OR 'aged'/exp OR 'middle aged'/exp OR adult*:ti,ab OR man:ti,ab OR men:ti,ab OR woman:ti,ab OR women:ti,ab)) | 4,520 |
| #3 | #1 AND #2 | 5,461 |
| #2 | 'mental disease'/exp OR 'suicidal behavior'/exp OR (mental NEAR/3 (dis* OR health)):ab,ti,kw OR (mental* NEAR/3 ill*):ab,ti,kw OR ((affective OR mood) NEAR/3 disorder*):ab,ti,kw OR depress*:ab,ti,kw OR anxiety:ab,ti,kw OR bipolar:ab,ti,kw OR PTSD:ab,ti,kw OR psychosis:ab,ti,kw OR psychotic:ab,ti,kw OR schizophren*:ab,ti,kw OR (post-traumatic NEAR/3 stress):ab,ti,kw OR (posttraumatic NEAR/3 stress):ab,ti,kw OR mental:ab,ti,kw OR suicid*:ab,ti,kw | 2,966,278 |
| #1 | 'refugee'/exp OR refugee*:ab,ti,kw OR (asylum NEAR/3 seeker*):ab,ti,kw OR (displaced NEAR/3 (person* OR people OR population* OR adult* OR female OR woman OR women OR male OR men OR men OR individual*)):ab,ti,kw OR stateless*:ab,ti,kw | 18,258 |

### APA PsycInfo (Ebsco) History February 4, 2021

| **Search** | **APA PsycInfo (Ebsco) Query – February 4, 2021** | **Items found** |
| --- | --- | --- |
| S4 | S3 NOT ((ZG ("adolescence (13-17 yrs)" OR "childhood (birth-12 yrs)" OR "infancy (2-23 mo)” OR "neonatal (birth-1 mo)" OR "preschool age (2-5 yrs)" OR "school age (6-12 yrs)") OR TI (adolescen* OR child* OR schoolchild* OR infant* OR girl* OR boy* OR teen OR teens OR teenager* OR youth* OR pediatr* OR paediatr* OR puber*) OR AB (adolescen* OR child* OR schoolchild* OR infant* OR girl* OR boy* OR teen OR teens OR teenager* OR youth* OR pediatr* OR paediatr* OR puber*)) NOT (ZG ("adulthood (18 yrs & older)" OR "aged (65 yrs & older)" OR "middle age (40-64 yrs)" OR "thirties (30-39 yrs)" OR "very old (85 yrs & older)") OR TI (adult* OR man OR men OR woman OR women) OR AB (adult* OR man OR men OR woman OR women))) | 3,606 |
| S3 | S1 AND S2 | 4,272 |
| S2 | DE ("Mental Health" OR "Mental Disorders" OR "Mental Health" OR "Psychiatric Symptoms" OR "Psychopathology" OR "Affective Disorders" OR "Bipolar Disorder" OR "Major Depression" OR "Affective Psychosis" OR "Schizoaffective Disorder" OR "Mania" OR "Posttraumatic Stress Disorder" OR "Post-Traumatic Stress" OR "Complex PTSD" OR "DESNOS" OR "Acute Stress Disorder" OR "Anxiety Disorders" OR "Generalized Anxiety Disorder" OR "Panic Disorder" OR "Schizophrenia" OR "Acute Schizophrenia" OR "Catatonic Schizophrenia" OR "Paranoid Schizophrenia" OR "Process Schizophrenia" OR "Schizophrenia (Disorganized Type)" OR "Schizophreniform Disorder" OR "Undifferentiated Schizophrenia" OR "Suicide" OR "Suicidal Ideation") OR TI (mental N3 dis* OR mental N3 ill* OR mentally N3 ill* OR mental N3 health OR affective N3 disorder* OR mood N3 disorder* OR depress* OR anxiety OR bipolar OR PTSD OR psychosis OR psychotic OR schizophren* OR post-traumatic N3 stress OR posttraumatic N3 stress OR mental OR suicid*) OR AB (mental N3 dis* OR mental N3 ill* OR mentally N3 ill* OR mental N3 health OR affective N3 disorder* OR mood N3 disorder* OR depress* OR anxiety OR bipolar OR PTSD OR psychosis OR psychotic OR schizophren* OR post-traumatic N3 stress OR posttraumatic N3 stress OR mental OR suicid*) OR KW (mental N3 dis* OR mental N3 ill* OR mentally N3 ill* OR mental N3 health OR affective N3 disorder* OR mood N3 disorder* OR depress* OR anxiety OR bipolar OR PTSD OR psychosis OR psychotic OR schizophren* OR post-traumatic N3 stress OR posttraumatic N3 stress OR mental OR suicid*) | 1,047,948 |
| S1 | DE ("Refugees" OR "Asylum Seeking" OR "Political Asylum") OR TI (refugee* OR asylum N3 seeker* OR (displaced N3 (person* OR people OR population* OR female OR woman OR women OR male OR man OR men OR individual* OR person* OR adult*)) OR stateless*) OR AB (refugee* OR asylum N3 seeker* OR (displaced N3 (person* OR people OR population* OR female OR woman OR women OR male OR man OR men OR individual* OR person* OR adult*)) OR stateless*) OR KW (refugee* OR asylum N3 seeker* OR (displaced N3 (person* OR people OR population* OR female OR woman OR women OR male OR man OR men OR individual* OR person* OR adult*)) OR stateless*) | 10,822 |

## **Quality Appraisal Checklist**

Three or more unclear or negative answers were considered to define a study with a high risk of bias.


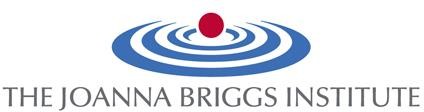


**JBI Critical Appraisal Checklist for Studies Reporting Prevalence Data -Modified**

Reviewer Author Year

|  | Yes | No | Unclear | |
| --- | --- | --- | --- | --- |
| 1. Were study participants recruited in an appropriate way?  Es: Studies may report random sampling from a population, and the methods section should report how sampling was performed. Random probabilistic sampling from a defined subset of the population (sample frame) should be employed in most cases, however, random probabilistic sampling is not needed when everyone in the sampling frame will be included/ analysed. For example, reporting on all the data from a good census is appropriate as a good census will identify everybody. When using cluster sampling, such as a random sample of villages within a region, the methods need to be clearly stated as the precision of the final prevalence estimate incorporates the clustering effect. Convenience samples, such as a street survey or interviewing lots of people at a public gatherings are not considered to provide a representative sample of the base population. | □ | □ | | □ |
| 2. Was the sample size adequate?  Es: The larger the sample, the narrower will be the confidence interval around the prevalence estimate, making the results more precise. An adequate sample size is important to ensure good precision of the final estimate. Ideally we are looking for evidence that the authors conducted a sample size calculation to determine an adequate sample size. This will estimate how many subjects are needed to produce a reliable estimate of the measure(s) of interest. For conditions with a low prevalence, a larger sample size is needed. Also consider sample sizes for subgroup (or characteristics) analyses, and whether these are appropriate. Sometimes, the study will be large enough (as in large national surveys) whereby a sample size calculation is not required. In these cases, sample size can be considered adequate | □ | □ | | □ |
| 3. Were the study subjects and the setting described in detail?  Es: Certain diseases or conditions vary in prevalence across different geographic regions and populations (e.g. Women vs. Men, sociodemographic variables between countries). The study sample should be described in sufficient detail so that other researchers can determine if it is comparable to the population of interest to them. | □ | □ | | □ |
| 4. Was the condition measured in a standard, reliable way for all participants?  Es: Considerable judgment is required to determine the presence of some health outcomes. Having established the validity of the outcome measurement instrument, it is important to establish how the measurement was conducted. Were those involved in collecting data trained or educated in the use of the instrument/s? If there was more than one data collector, were they similar in terms of level of education, clinical or research experience, or level of responsibility in the piece of research being appraised? When there was more than one observer or collector, was there comparison of results from across the observers? Was the condition measured in the same way for all participants? | □ | □ | | □ |
| 5. Was the response rate adequate, and if not, was the low response rate managed appropriately? | □ | □ | | □ |

Es: A large number of dropouts, refusals or “not founds” amongst selected subjects may diminish a study’s validity, as can a low response rates for survey studies. The authors should clearly discuss the response rate and any reasons for non-response and compare persons in the study to those not in the study, particularly with regards to their socio-demographic characteristics.

# © Joanna Briggs Institute 2017 Critical Appraisal Checklist for Prevalence Studies

**Quality of included studies**

| Author | Item1* | Item2* | Item3* | Item4* | Item5* | Quality |
| --- | --- | --- | --- | --- | --- | --- |
| Ainamani et al. (2020) | no | no | yes | yes | no | High |
| Akinyemi et al. (2012) | yes | yes | no | no | yes | Low |
| Bapolisi et al. (2020) | yes | yes | yes | no | yes | Low |
| Bhui et al. (2006) | yes | no | yes | yes | no | Low |
| Bogic et al. (2012) | no | yes | yes | no | no | High |
| Cheung et al. (1994) | yes | yes | unclear | yes | yes | Low |
| Civan et al. (2020) | no | no | yes | yes | yes | Low |
| Eckart et al. (2011) | no | no | yes | yes | no | High |
| Fenta et al. (2004) | yes | no | yes | no | no | High |
| Heeren et al. (2012) | no | no | yes | no | no | High |
| Hinton et al. (1993) | unclear | unclear | yes | no | yes | High |
| Hocking et al. (2015) | no | no | yes | unclear | unclear | High |
| Hocking et al. (2018) | yes | unclear | yes | unclear | unclear | High |
| Jakobsen et al. (2011) | no | no | yes | yes | no | Low |
| Kaur et al. (2020) | yes | yes | yes | no | yes | Low |
| Kazour et al. (2017) | no | no | yes | unclear | yes | High |
| Kizilhan et at. (2018) | no | no | yes | yes | no | HIgh |
| Laban et al.(2004) | yes | yes | yes | yes | yes | Low |
| Llosa et al. (2014) | yes | yes | yes | no | yes | Low |
| Maier et al. (2010) | no | no | yes | no | no | High |
| Marshall et al. (2005) | yes | yes | yes | no | yes | Low |
| Momartin et al. (2004) | no | no | yes | yes | yes | Low |
| Naja et al. (2016) | no | no | yes | no | yes | High |
| Nosè et al. (2018) | yes | no | yes | no | yes | Low |
| Rasmussen et al. (2012) | yes | yes | yes | no | no | Low |
| Rees et al. (2019) | yes | yes | yes | yes | yes | Low |
| Renner et al. (2006) | no | no | yes | no | no | High |
| Richter et al. (2018) | no | no | yes | no | no | High |
| Sagaltici et al. (2020) | no | yes | yes | no | no | High |
| Segal et al. (2018) | no | no | yes | no | yes | High |
| Steel et al. (2002) | yes | yes | yes | no | yes | Low |
| Sundvall et al. (2020) | no | no | yes | yes | no | High |
| Tay et al. (2013) | yes | no | yes | yes | yes | Low |
| Tekeli-Yesil et al. (2018) | no | no | yes | no | no | High |
| Tekin et al. (2016) | yes | yes | yes | no | yes | Low |
| Turner et al. (2003) | no | no | yes | no | yes | High |
| Van Ommeren et al. (2001) | yes | yes | yes | yes | yes | Low |
| von Lersner et al. (2008) | no | no | yes | yes | yes | Low |
| Wright et al. (2017) | yes | yes | yes | yes | no | Low |
| Wulfes et al. (2019) | no | no | yes | yes | no | High |

*Item1: Were study participants recruited in an appropriate way?; Item2: Was the sample size adequate?; Item3: Were the study subjects and the setting described in detail?; Item4: Was the condition measured in a standard, reliable way for all participants?; Item5: Was the response rate adequate, and if not, was the low response rate managed appropriately?

**Supplementary forest plots: MDD and PTSD without outliers and subgroups (by disorders)**


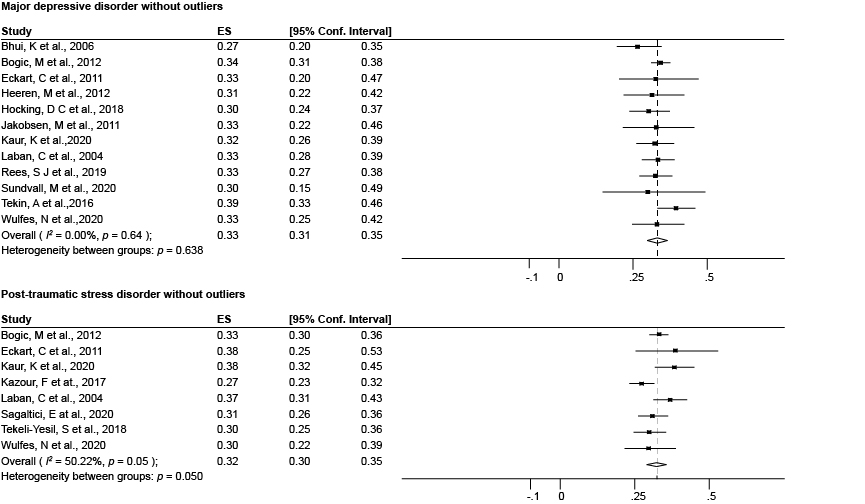

≥5: people who have spent more than five years as refugees or asylum seekers; >1<5: people who have spent between 5 years and 1 year as refugees or asylum seekers; <1: people who have spent less then 1 year as refugees or asylum seekers

Other diagnostic interviews: CIDI: Composite International Diagnostic Interview; DIS: Diagnostic Interview Schedule; SCID: The Structured Clinical Interview for DSM);

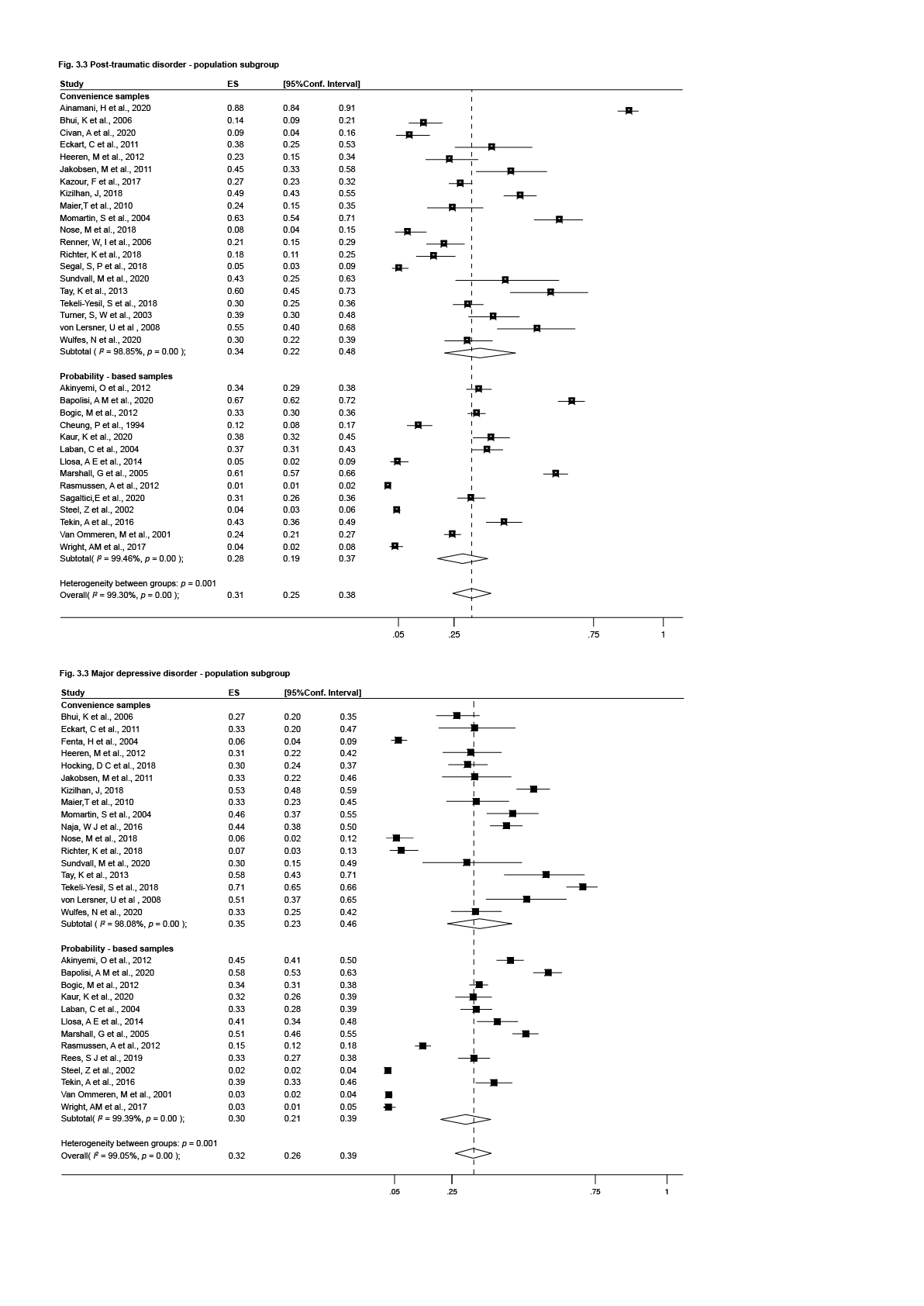

HICs: High income countries; LMICs: Low middle income countries

Other diagnostic interviews: CAPS: Clinician-Administered PTSD Scale; CIDI: Composite International Diagnostic Interview; DIS: Diagnostic Interview Schedule; SCID: The Structured Clinical Interview for DSM.

≥5: people who have spent more than five years as refugees or asylum seekers; >1<5: people who have spent between 5 years and 1 year as refugees or asylum seekers; <1: people who have spent less then 1 year as refugees or asylum seekers
